# Supplementary material for: Molecular Epidemiology of Plasmid-Mediated Types 1 and 3 Fimbriae Associated with Biofilm Formation in Multidrug Resistant Escherichia coli from Diseased Food Animals in Guangdong, China
Source: Microbiol Spectr. 2022 Aug 15;10(5):e02503-21. doi: 10.1128/spectrum.02503-21 (PMC9603762; doi:10.1128/spectrum.02503-21)
Supplement: Supplemental file 1 — Supplemental material. Download spectrum.02503-21-s0001.pdf, PDF file, 0.3 MB [file spectrum.02503-21-s0001.pdf]

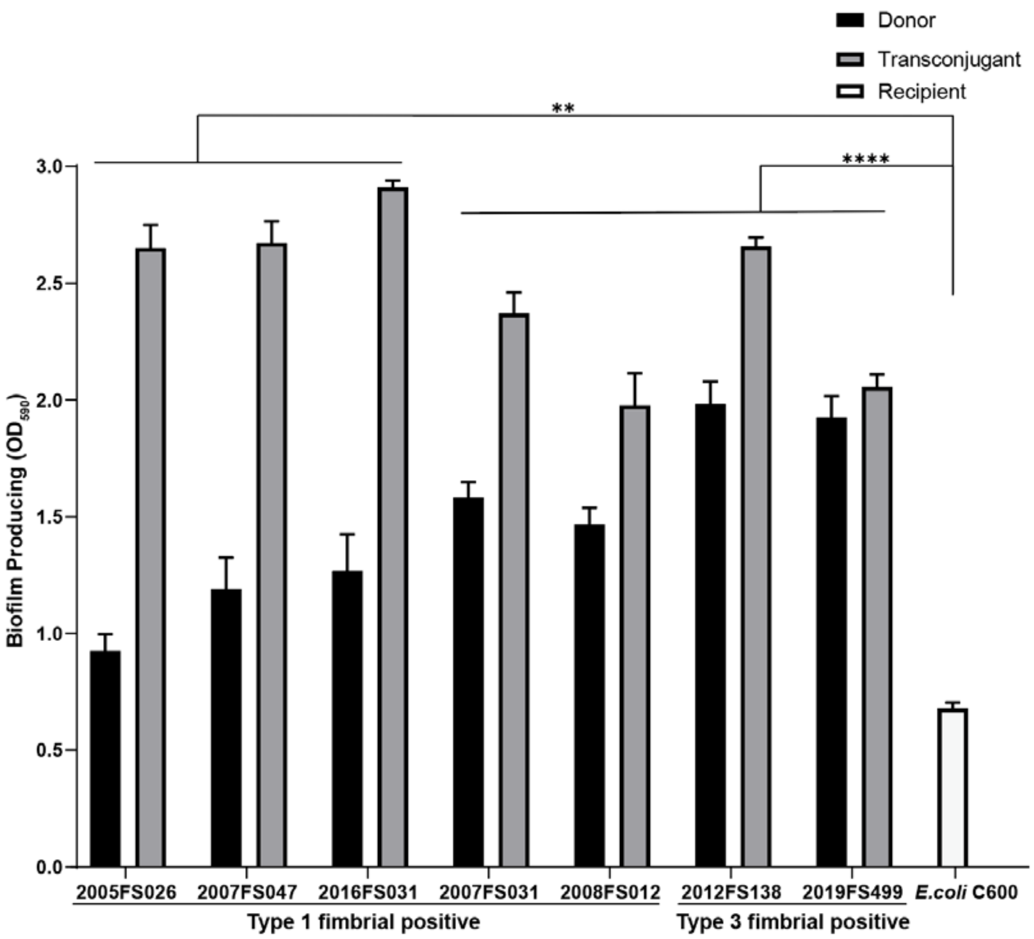

Figure S1 Quantification of biofilm biomass by crystal violet staining. The mean values and standard deviations of three biologically independent samples are shown. **, $P < 0.01$; ****, $P < 0.0001$ tested by one-way analysis of variance.

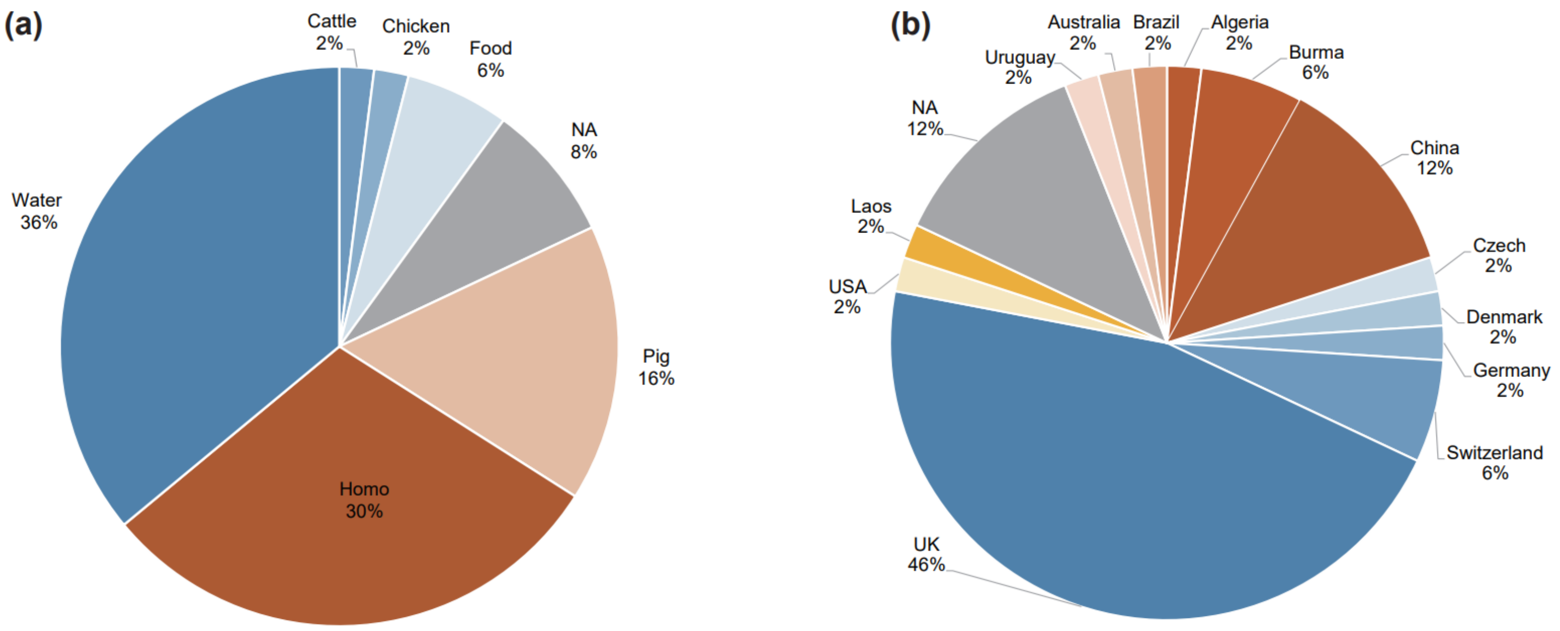

Figure S2 The origin and location distribution of 50 *mrkABCDF* operons from the GenBank data archive. (a) origin distribution; (b) location distribution.

Table S1 Sequences of primers used in this study

Primer	Sequence (5’-3’)	Description
control-test-F	CACGACTTATCGCCACTGG	Detection of Vector plasmid pMD19-T
control-test-R	CTGCGTGCAATCCATCTTGTTC	
fim-test-F	CACGACTTATCGCCACTGG	Detection of the *fimACDH* operon
fim-test-R	GTAAAGTCTGCGCTTTTACCGC	
mrk-test-F	CACGACTTATCGCCACTGG	Detection of the *mrkABCDF* operon
mrk-test-R	CGGGTATCTGGCCAATACCTTG	
fim-F1	GCTTAATCAGTGAGGCACCTATCTCTCGCTGTCGTTTTCAAAATC	Construction of the *fimACDH* operon clone in expression vector pMD19-T
fim-R1	TACTGGATCTATCAACAGGAGTCTCAGGGGTAGATAATATTCAGC	
fim-F2	GCTGAATATTATCTACCCCTGAGACTCCTGTTGATAGATCCAGTA	
fim-R2	GATTTTGAAAACGACAGCGAGAGATAGGTGCCTCACTGATTAAGC	
mrk-F1	GGGCAGCATCACCGGAATTCGTCGCCGATGATATTGCCATG	Construction of the *mrkABCDF* operon clone in expression vector pMD19-T
mrk-R1	TACTGGATCTATCAACAGGAGTCCAGAGCAGAGATAGCGCTGATG	
mrk-F2	CATCAGCGCTATCTCTGCTCTGGACTCCTGTTGATAGATCCAGTA	
mrk-R2	GAATTCCGGTGATGCTGCCCTGTCAGACCAAGTTTACTCAC	

Table S2 Antimicrobial susceptibility testing of wild type and clone strains carrying *fimACDH* or *mrkABCDF* operons

Strain	OD_{590}	MICs (mg/L)														
		AMP	CIF	AMK	GEN	APR	STR	TET	DOX	FOS	FFL	CST	CIP	S/T	OLA	MEM
**2005FS026(*fimACDH* wild type)**	1.05±0.05	> 256	0.5	1	64	8	128	> 256	64	2	256	0.5	32	160	256	0.015
E. coli* DH5α-PMD™19-T-*fimACDH	1.48±0.19	0.125	0.125	1	0.125	2	0.5	0.5	0.5	0.5	0.25	0.125	0.004	0.3	0.25	0.015
**2010FS332((*mrkABCDF* wild type)**	2.19±0.06	> 256	0.25	8	16	> 256	256	> 256	16	2	256	16	> 256	160	256	0.015
E. coli* DH5α-PMD™19-T-*mrkABCDF	2.11±0.03	0.125	0.25	1	0.25	4	1	0.5	1	2	0.25	0.25	0.004	5	0.25	0.015
***E. coli* DH5α-empty-Vector**	0.68±0.10	0.125	0.125	1	0.125	2	0.5	0.5	0.5	0.5	0.25	0.125	0.004	0.3	0.25	0.015

AMP, ampicillin; CIF, ceftiofur; AMK, amikacin; GEN, gentamicin; APR, apramycin; STR, streptomycin; TET, tetracycline; DOX, doxycycline; FOS, fosfomycin; FFL, florfenicol; CST, colistin; CIP, ciprofloxacin; S/T, trimethoprim/sulfamethoxazole; OLA, olaquindox; MEM, Meropenem.

Table S3 The detection rates of *fimACDH* and *mrkABCDF* operons among 6 Enterobacteriaceae members

Strain	The detection rate of *fim*-operon	The detection rate of *mrk*-operon
**This study**		
Escherichia coli	7/123 (5.7%)	43/123 (34.96%)
**NCBI Database**		
Escherichia coli	60/21387 (0.28%)	199/21387 (0.62%)
Klebsiella pneumoniae	8/9457 (0.08%)	9457/9457 (%)
Enterobacter cloacae	133/214 (62.15%)	14/214 (6.54%)
Citrobacter freundii	1/330 (0.3%)	13/330 (3.94%)
Salmonella spp.	0/12535 (0)	0/12535 (0)
Proteus mirabilis	0/265 (0)	0/265 (0)

Materials and methods

Biofilm formation assay and antimicrobial susceptibility testing

Quantification of static biofilm producing in 96-well flat-bottom polystyrene microtiter plate by using crystal violet staining. The extent of biofilm formation was determined using the classification method as previously described (1). The cutoff value (OD) for this classification was defined as 3 standard deviations above the mean OD_{590} of the negative control. Bacterial isolates were classified into 3 categories; weak and none ($OD_{590} \leq 2 \times$ control), moderate ($2 \times$ control $< OD_{590} \leq 4\times$ control), and strong ($OD_{590} > 4 \times$ control). All moderate-to-strong biofilm producers were identified by matrix-assisted laser desorption/ionization-time-of-flight mass spectrometry and 16S rRNA gene sequence-based analyses.

Antimicrobial susceptibilities of the tested isolates were determined by the agar dilution method and the results were interpreted according to the Clinical and Laboratory Standards Institute (CLSI, 2018: M100-S28) (2), veterinary CLSI (VET01-A4E/VET01-S3E) (3). The following antimicrobials were tested: ampicillin (AMP), ceftiofur (CIF), amikacin (AMK), gentamicin (GEN), apramycin (APR), tetracycline (TET), doxycycline (DOX), florfenicol (FFL), ciprofloxacin (CIP), olaquindox (OLA), trimethoprim/sulfamethoxazole (S/T), fosfomycin (FOS), colistin (CST), and meropenem (MEM). *E. coli* ATCC 25922 was used as the quality control strain.

Conjugation assays

Transconjugants were selected on MacConkey agar plates supplemented with florfenicol (8 mg/L) or colistin (16 mg/L) and streptomycin (1500 mg/L). Antimicrobial susceptibility testing was conducted on transconjugants and the *mrkD* and *fimD* genes were confirmed by PCR. PCR-based replicon typing and pMLST were performed for transconjugants as previously described (4, 5).

Scanning Electron Microscopy (SEM)

Biofilms were grown on borosilicate glass coverslips and in the wells of a

6-well polystyrene microtiter plates. The coverslips were washed twice with PBS (pH 7.4) and then fixed with glutaraldehyde (2.5%) for 2 h at 4°C. The coverslips were then immersed in an ethanol gradient (30-90%) for 10 min per concentration. The glass slides were covered with gold after drying in a critical point dryer and the aggregated biofilms were examined by scanning electron microscopy (6).

Detecting the type 1/3 fimbriae operon genes and its phylogenetic relationship

To understand the spread of the *mrkABCDF*/*fimACDH* gene clusters, all strong biofilm *E. coli* producers were screened for these two operons by PCR (Table S1). The prevalence of the *mrk*/*fim* operons were further investigated among Enterobacterium with WGS data from publicly available databases (https://www.ncbi.nlm.nih.gov/datasets) by searching against the *mrk*/*fim* operons from 2010FS332/2005FS026 with ≥ 70% nucleotide identity and ≥ 90% query coverage.

To further understand the distribution and phylogenetic relationship of *mrk*/*fim* operons among Enterobacteriaceae, the concatenated *mrk*/*fim* operons from GenBank were further selected by searching against the *mrk*/*fim* operons from 2010FS332/2005FS026 with ≥ 80% nucleotide identity and ≥ 95% query coverage. Phylogenetic correlation between the concatenated *mrk*/*fim* operons in this study and those from GenBank were conducted by building Maximum likelihood (ML) trees based on amino acid sequences.

References

1. Nielsen DW, Klimavicz JS, Cavender T, Wannemuehler Y, Barbieri NL, Nolan LK, Logue CM. 2018. The Impact of Media, Phylogenetic Classification, and *E. coli* Pathotypes on Biofilm Formation in Extraintestinal and Commensal *E. coli* From Humans and Animals. Front Microbiol 9:902.
2. Clinical and Laboratory Standards Institute. 2018. Performance standards for antimicrobial susceptibility testing; 28th informational supplement. CLSI document M100-S28. Clinical and Laboratory Standards Institute, Wayne, PA.
3. Clinical and Laboratory Standards Institute. 2015. Performance standards for antimicrobial disk and dilution susceptibility tests for bacteria isolated from animals; approved standard—fourth edition and supplement, VET01-A4E and VET01-S3E. Clinical and Laboratory Standards Institute, Wayne, PA.

4. Carattoli A, Bertini A, Villa L, Falbo V, Hopkins KL, Threlfall EJ. 2005. Identification of plasmids by PCR-based replicon typing. J Microbiol Methods 63:219-28.
5. Hancock SJ, Phan MD, Peters KM, Forde BM, Chong TM, Yin WF, Chan KG, Paterson DL, Walsh TR, Beatson SA, Schembri MA. 2017. Identification of IncA/C Plasmid Replication and Maintenance Genes and Development of a Plasmid Multilocus Sequence Typing Scheme. Antimicrob Agents Chemother 61.
6. Ribeiro KVG, Ribeiro C, Dias RS, Cardoso SA, de Paula SO, Zanuncio JC, de Oliveira LL. 2018. Bacteriophage Isolated from Sewage Eliminates and Prevents the Establishment of *Escherichia Coli* Biofilm. Adv Pharm Bull 8:85-95.
